# CXCL10 is a prognostic marker for pancreatic adenocarcinoma and tumor microenvironment remodeling

**DOI:** 10.1186/s12885-023-10615-w

**Published:** 2023-02-13

**Authors:** Yuan Nie, Chao Liu, Qi Liu, Xuan Zhu

**Affiliations:** Department of Gastroenterology, Jiangxi Clinical Research Center for Gastroenterology, The First Affiliated Hospital of Nanchang University, Yongwaizhengjie Road, 330006 Donghu District Nanchang, Jiangxi China

**Keywords:** CXCL10, Pancreatic adenocarcinoma, Tumor microenvironment, Immune infiltration, Prognosis

## Abstract

**Background:**

The tumor microenvironment (TME) plays a crucial role in the progression of pancreatic adenocarcinoma (PAAD). However, challenges remain regarding the role played by TME associated genes in the prognosis of PAAD.

**Methods:**

The scores of tumor infiltrating immune cells (TICs), the immune and stroma scores of 182 PAAD patients in the Cancer Genome Atlas (TCGA) database were determined using CIBERSORT and ESTIMATE calculations. The final genes were identified by protein-protein interaction (PPI) networks and univariate Cox regression of differentially expressed genes. Finally, the correlation between gene expression and TCGA and clinical characteristics of patients in local hospital database was discussed. Gene set enrichment analysis (GSEA), the association between CXCL10 expression and TICs components were conducted.

**Results:**

In TCGA database and local hospital data, CXCL10 expression was correlated with the survival rate and TNM classification of patients with PAAD. Immune-related activities were enriched in the CXCL10 high expression group, while metabolic pathways were enriched in the CXCL10 low expression group. The expression of CXCL10 correlated with the proportion of TICs. CXCL10 expression was correlated with the proportion of TICs.

**Conclusion:**

CXCL10 is a potential prognostic marker for PAAD and provide additional insights into the treatment of PAAD based on TME transformation. However, more independent experimentation with the CXCL10 is need.

**Supplementary Information:**

The online version contains supplementary material available at 10.1186/s12885-023-10615-w.

## Introduction

Pancreatic adenocarcinoma (PAAD) is one of the most lethal malignant tumors in the world and is characterized by late detection, difficult treatment and poor prognosis. The 5-year survival rate of PAAD patients after surgical resection, radiotherapy and chemotherapy is still less than 10% [1]. Because of the close relationship between the tumor microenvironment (TME) and the tumorigenesis/progression of PAAD, exploring the carcinogenesis and therapeutics of PAAD is urgent [2]. In recent years, based on the theory of TME regulation, immunotherapy has become an emerging cancer treatment strategy. The TME refers to the interaction between tumor cells and the surrounding tissue components that forms a complex internal environment that is conducive to the biological behavior of tumor cells [3]. The TME usually consists of matrix components, cellular components, and soluble cytokines. The TME of pancreatic cancer has the following characteristics: a large number of compact matrix components, such as the activated proliferation of pancreatic stellate cells, tumor-related fibroblasts, type I collagen, hyaluronic acid and other extracellular matrix components; various types of immune cells of innate immunity and adaptive immunity; and a large number of soluble immunosuppressive factors [4, 5]. Previous studies have shown that the tumor-infiltrating immune cells (TICs) in the TME play an important role in the development of PAAD and serve as a predictive parameter of prognosis [6]. In patients with pancreatic cancer, the numbers of CD4 + and CD8 + effector T cells, natural killer (NK) cells and dendritic cells (DCs) are usually reduced and present nonfunctional or immature phenotypes and states, while regulatory T cells often exhibit immunosuppressive effects and exist in large numbers [6]. Previous studies have shown that the number of Treg cells in the TME is positively correlated with the progression and poor prognosis of PAAD [7]. PAAD is rich in dense matrix and TICs with immunosuppressive effects, which creates favorable conditions for the occurrence, development and distant metastasis of PAAD. Therefore, the analysis of TICs in PAAD is helpful for studying its pathogenesis.

CXC chemokine ligand 10 (CXCL10) is a kind of lymphocyte chemotactic protein produced by interferon-γ (IFN-γ) or lipopolysaccharide (LPS), also known as interferon-induced protein 10 (IP-10). Chemokine CXC receptor 3 (CXCR3) is the only receptor of CXCL10. It has been reported that CXCL10 is associated with the occurrence, development, therapeutic efficacy and prognosis of various tumors [8]. CXCL10 mainly activates downstream signaling pathways (including MAPK and PI3K/Akt) by binding to CXCR3 and then produces biological effects [9]. CXCL10 can combine with CXCR3 to mediate chemotaxis and confer anti-apoptosis and pro-proliferation effects. CXCL10 can also bind to the TLR4 receptor to activate protein kinase B (PKB) and c-Jun N-terminal kinase (JNK), leading to the activation of caspase-8 and caspase-3 and causing cell lysis [10]. CXCL10 plays an important role in the regulation of the TME by recruiting CXCR3-positive T cells, B cells, monocytes/macrophages, DCs and NK cells. Here, we explored the differentially expressed genes (DEGs) generated by comparing immune components and stromal components in PAAD samples and revealed that CXCL10 might be a potential indicator for the alteration of TME status in PAAD.

## Materials and methods

### Raw data

The transcriptome RNA data of 182 samples (4 normal and 178 tumor samples) and clinical information were downloaded from TCGA (https://portal.gdc.cancer.gov/). Meanwhile, the blood serum from PAAD patients or healthy individuals in local hospital were collected in this study. Exclusion criteria: refused consent, cerebrovascular disease, cardiovascular disease, hematological disease, renal failure, other cancer. Refusal to give consent, cerebrovascular disease, cardiovascular disease, hematologic disorders, renal failure, the simultaneous presence of other cancer and corresponding treatment were the exclusion criteria (Supplementary Fig. 1). The study protocol was met with the declaration of Helsinki and was approved by the Institutional Ethics Committee of First Affiliated Hospital of Nanchang University (No. 2017 − 0106).

### Generation of the differentially expressed genes

Three scoring forms (immune score, stromal score, and ESTIMATE score) in the TME were calculated using the Feat estimation algorithm. The PAAD patients were labeled as high or low group based on the median of immune score, stromal score, and ESTIMATE score. DEGs were generated by comparing high-score and low score samples using the limma package. Gene Ontology (GO) and Kyoto Encyclopedia of genes and genomes (KEGG) Enrichment analysis was performed by clusterprofiler, enrichplot, and ggplot2 packages. Only terms with both p-value and Q-value < 0.05 were considered significantly enriched. Only pathways with both P-value and Q-value < 0.05 were considered significantly enriched.

### Screening process of the final gene

The protein-protein interaction (PPI) network was constructed by the Search Tool for the Retrieval of Interacting Genes/Proteins database, which conducted by Cystoscope version 3.6.1. Nodes with an interaction confidence larger than 0.95 were used to construct the network. The univariate Cox regression was performed using the survival package in R language and the top 18 genes ranked by Q-value are shown in the figure. Hazardous rate: the percentage of events occurring in a unit time to the total number of subjects. Hazardous ratio: the ratio of two risk rates, usually referring to the ratio between the experimental group and the control group. Survival analysis was conducted by the survminer packages in the R language. The Kaplan-Meier (K-M) method was used to plot survival curves.

### ELISA and real-time quantitative PCR

Serum CXCL10 levels were measured by ELISA. PCR was performed with a reaction mixture containing cDNA template, and TB Green™ Fast qPCR Mix in a Step One Plus Real-Time PCR System (Thermo Fisher Scientific). The primers of CXCL10 were 5’-GTACGCTGTACCTGCATCAGCATTAG-3’ and 5’-CTGGATTCAGACATCTCTTCTCACCC-3’. The relative abundances of the target genes were determined by standard curve and the GAPDH was set as loading control.

### Differential expression of genes and clinical characteristics

SPSS software version 16.0 (SPSS, Chicago) and R3.62 were used to conducted in statistical analysis. Continuous and categorical variables were described as median (interquartile range [IQR]) and percentage (%). P < 0.05 was considered statistically significant. P < 0.05 was considered statistically significant.

### Gene set enrichment analysis (GSEA)

The hallmark collection was downloaded from the molecular signatures database as the target set for GSEA using GSEA 3.0 software. The transcriptome of all PAAD patients was used for GSEA, and only gene sets with NOM P < 0.05 and FDR Q < 0.05 were considered significant.

### TIC profile

The TIC abundance distributions of all PAAD patients were determined by the CIBERSORT calculation method, and only PAAD samples with P < 0.05 were selected for the following analysis. The TIC abundance profiles of all PAAD patients were determined by the CIBERSORT computational method, and only PAAD patients with P < 0.05 were selected for the in-depth analysis.

## Results

### Scores were associated with the clinical characteristics of PAAD patients

**Fig. 1:**
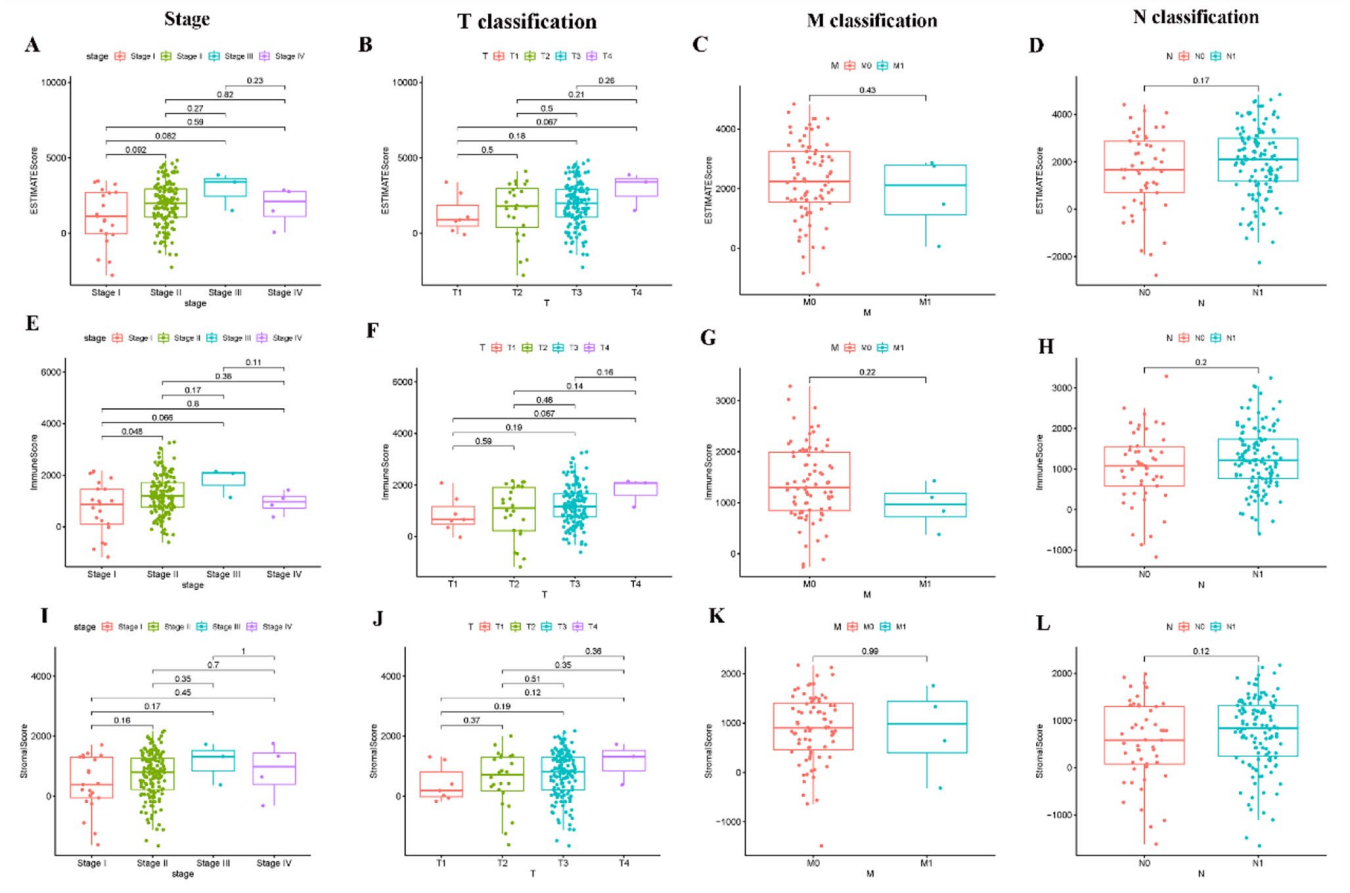
Correlation of ESTIMATE Score, Immune Score and Stromal Score with clinical characteristics. (A–D) Distribution of ESTIMATE Score in different stage and TMN classification; (E–H) Distribution of Immune Score in different stage and TMN classification; (I–L) Distribution of Stromal Score in different stage and TMN classification

The transcriptome RNA-seq data of 182 patients were downloaded from the TCGA database followed by calculation with the CIBERSORT and ESTIMATE algorithms. The clinical information of PAAD patients from TCGA was collected (Supplementary Table 1). In the comparison of immune score, the score of stage II patients were higher than those of stage I patients and lower than those of stage III patients; the score of the T2 classification of TNM stage were higher than those of the T1 classification and were lower than those of the T3 classification; the score of the N1 classification of TNM stage were higher than those of the T0 classification (Fig. 1E-H). Similarly, similar trend results can also be found in comparing of stromal score (Fig. 1I-L). Although not statistically significant, the ESTIMATE scores are also shown to increase with stage, T classification of TNM stage, N classification of TNM stage (Fig. 1A-D). By analyzing the 22 PAAD patients from the local hospital. the ESTIMATE score, immune score, and stromal score of stage also shows some higher trends (Supplementary Fig. 2). These results suggested that the TME was associated with the progression of PAAD, especially the immune-related tumor microenvironment.

### DEGs between lower and higher immune scores and stromal scores

**Fig. 2:**
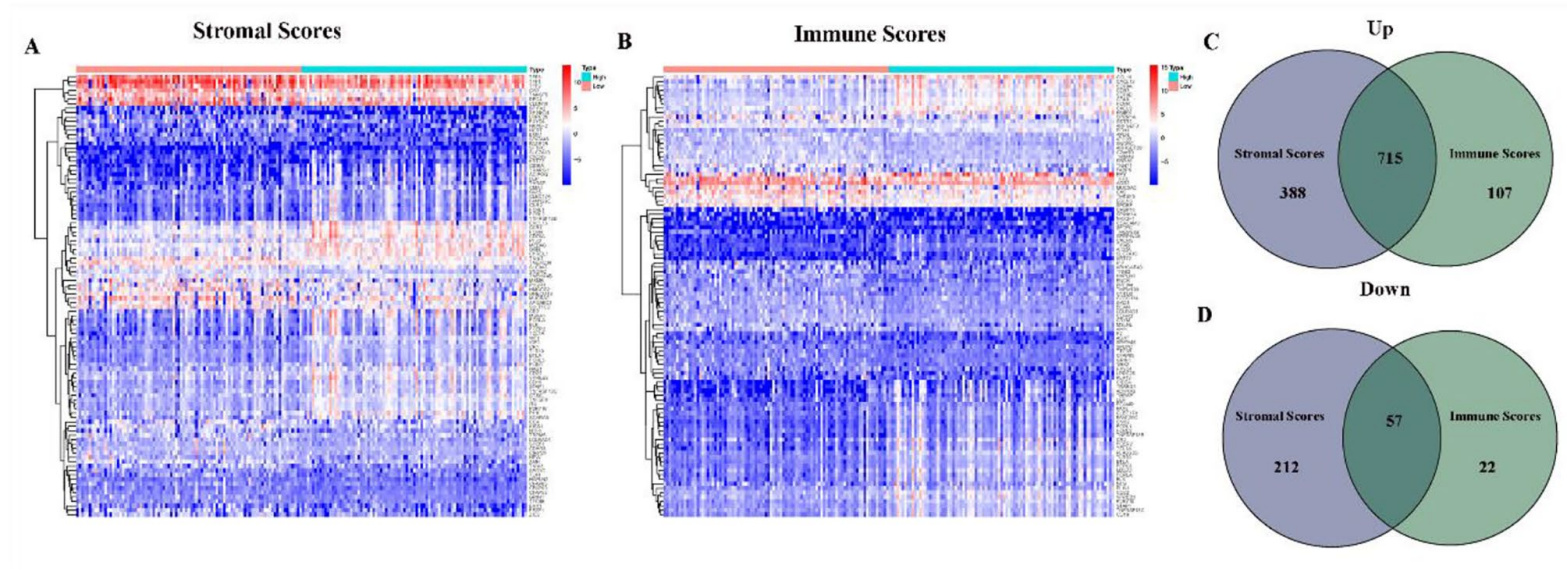
Heatmaps and Venn plots for DEGs. (A) Heatmap for DEGs generated by comparison of the high score group vs. the low score group in Stromal Score; (B) Heatmap for DEGs generated by comparison of the high score group vs. the low score group in Immune Score; (C, D) Venn plots showing common up-regulated and down-regulated DEGs shared by Immune Score and Stromal Score

In order to determine differences in gene expression, gene expression in the high and low score samples was compared and analyzed as shown in Fig. 2. A total of 600 DEGs were obtained based on matrix scores (high score samples versus low score samples) compared to the median (Fig. 2A-B). Similarly, a cross analysis of 129 DEG Venn diagrams based on immune scores showed that a total of 715 up-regulated genes scored high in immune and stromal scores, while 57 down-regulated genes scored low (Fig. 2C-D).

### GO and KEGG enrichment analysis

**Fig. 3:**
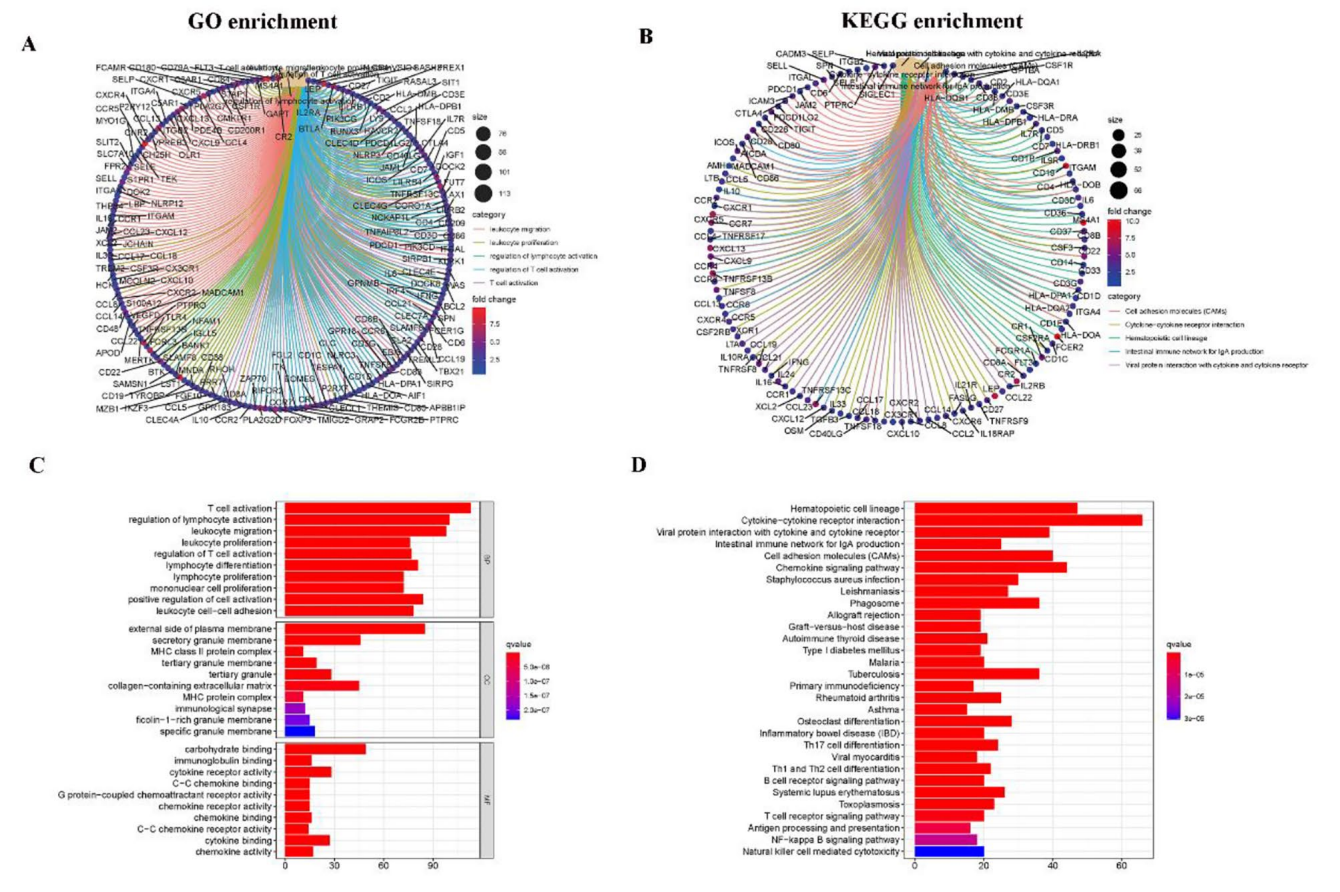
Enrichment analysis of GO and KEGG for DEGs. (A, C) GO enrichment analysis for 772 DEGs; (B, D) KEGG enrichment analysis for 772 DEGs

As shown in Fig. 3, GO enrichment analysis results showed that DEG mainly corresponded to immune-related GO terms, such as T cell activation, lymphocyte activation regulation, and leukocyte migration (Fig. 3A C). KEGG enrichment analysis also showed enrichment of T cell activation and regulation of lymphocyte activation (Fig. 3B and D). Thus, the overall function of DEG appears to be related to immune-related activities, suggesting that the involvement of immune factors is a major feature of TME in PAAD.

### Intersection analysis of the PPI network and univariate Cox regression

**Fig. 4:**
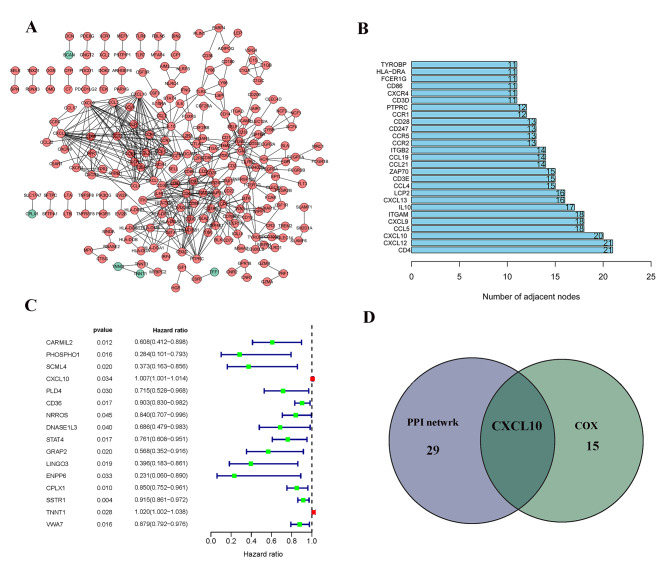
Protein–protein interaction network and univariate COX. (A) Interaction network constructed with the nodes with interaction confidence value > 0.95; (B)The top 30 genes ordered by the number of nodes; (C) Univariate COX regression analysis with 772 DEGs, listing the top significant factors with P < 0.005; (D) Venn plot showing the common factors shared by leading 30 nodes in PPI and top significant factors in univariate COX

To further explore the underlying mechanism, Cytoscape software was used to build a PPI network based on a string database. The interactions are shown in Fig. 4A, and the bar graph represents the top 30 genes by node number (Fig. 4B). Univariate Cox regression analysis was used to determine the important factors affecting the survival rate of PATIENTS with PAAD (Fig. 4C). Then, the leading nodes in the PPI network and the top 16 factors ranked by univariate Cox regression P value were cross-analyzed, and only one factor, CXCL10, overlapped with the above analysis (Fig. 4D). Therefore, CXCL10 was the final gene to be screened and further analyzed.

### Correlations of CXCL10 with the clinical characteristics of PAAD patients from the TCGA and hospital databases

**Fig. 5:**
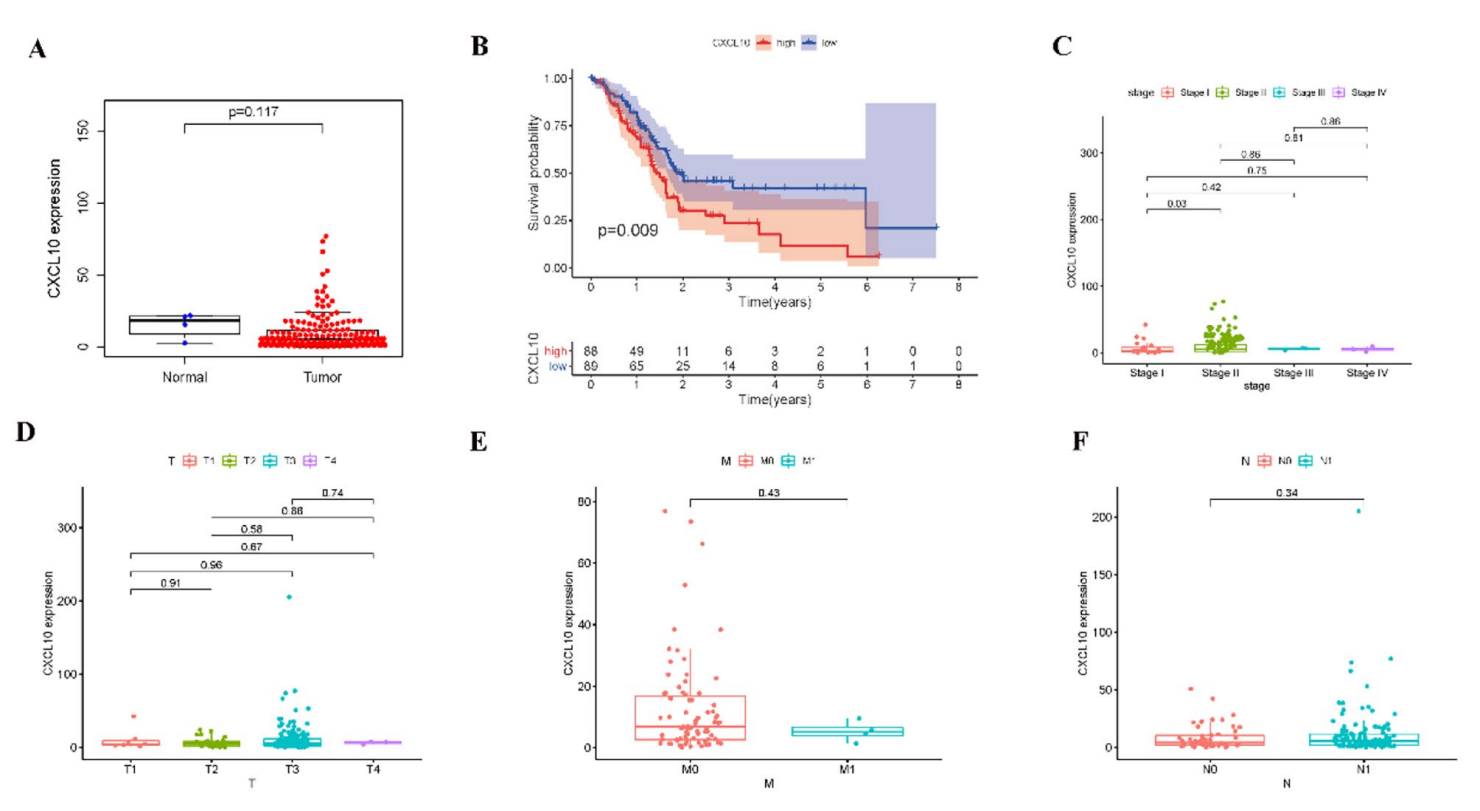
The differentiated expression of CXCL10 and correlation with survival and clinical characteristics. (A) Differentiated expression of CXCL10 in the normal and tumor sample; (B) Survival analysis for PAAD patients with different CXCL10 expression; (C–F) The correlation of CXCL10 expression with clinical characteristics

**Fig. 6:**
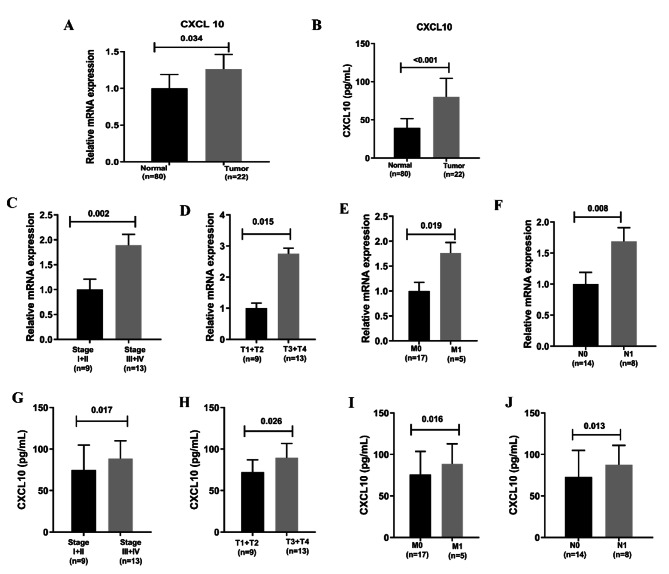
The CXCL10 level and correlation with clinical characteristics. (A) The relative mRNA expression of CXCL10 in normal patients, PAAD patients; (B) The CXCL10 level by ELISA in normal patients, PAAD patients; (C–F) The correlation of CXCL10 mRNA expression with clinical characteristics; (G–J) The correlation of CXCL10 level by ELISA with clinical characteristics

The expression of CXCL10 in normal tissues and pancreatic cancer tissues were compared based on TCGA databases. It may be due to the small amount of normal tissue samples; the results did not show significant statistical difference (Fig. 5A). According to the gene expression of CXCL10, all PAAD samples were grouped into a high-expression group and a low-expression group. The survival analysis showed that PAAD patients with lower expression levels had longer survival times than those with higher expression levels (Fig. 5B). In the comparison of stage, the expression level of CXCL10 of stage II patients were significantly higher than those of stage I patients (Fig. 5C). Considering the limited number of PAAD cases in different stages or TNM stages, there were no significant differences in CXCL10 gene expression, but the increasing trend is shown in Fig. 5D-F. There were 102 patients, including 80 normal patients and 22 tumor patients. In the paired analysis of local hospital database, the expression level of CXCL10 in the tumor samples was significantly higher than that in the normal samples (Fig. 6A-B). By applying the quantitative PCR, the expression of CXCL10 in relatively advanced tumors (stage III + IV, T3 + T4, M1, N1) was significantly higher than that in relatively early tumors (stage I + II, T1 + T2, M0, N0) (Fig. 6C-F). By applying the ELISA, the level of CXCL10 in relatively advanced tumors was significantly higher than that in relatively early tumors (Fig. 6G-J).

### CXCL10 has potential to be an indicator of TME modulation

**Fig. 7:**
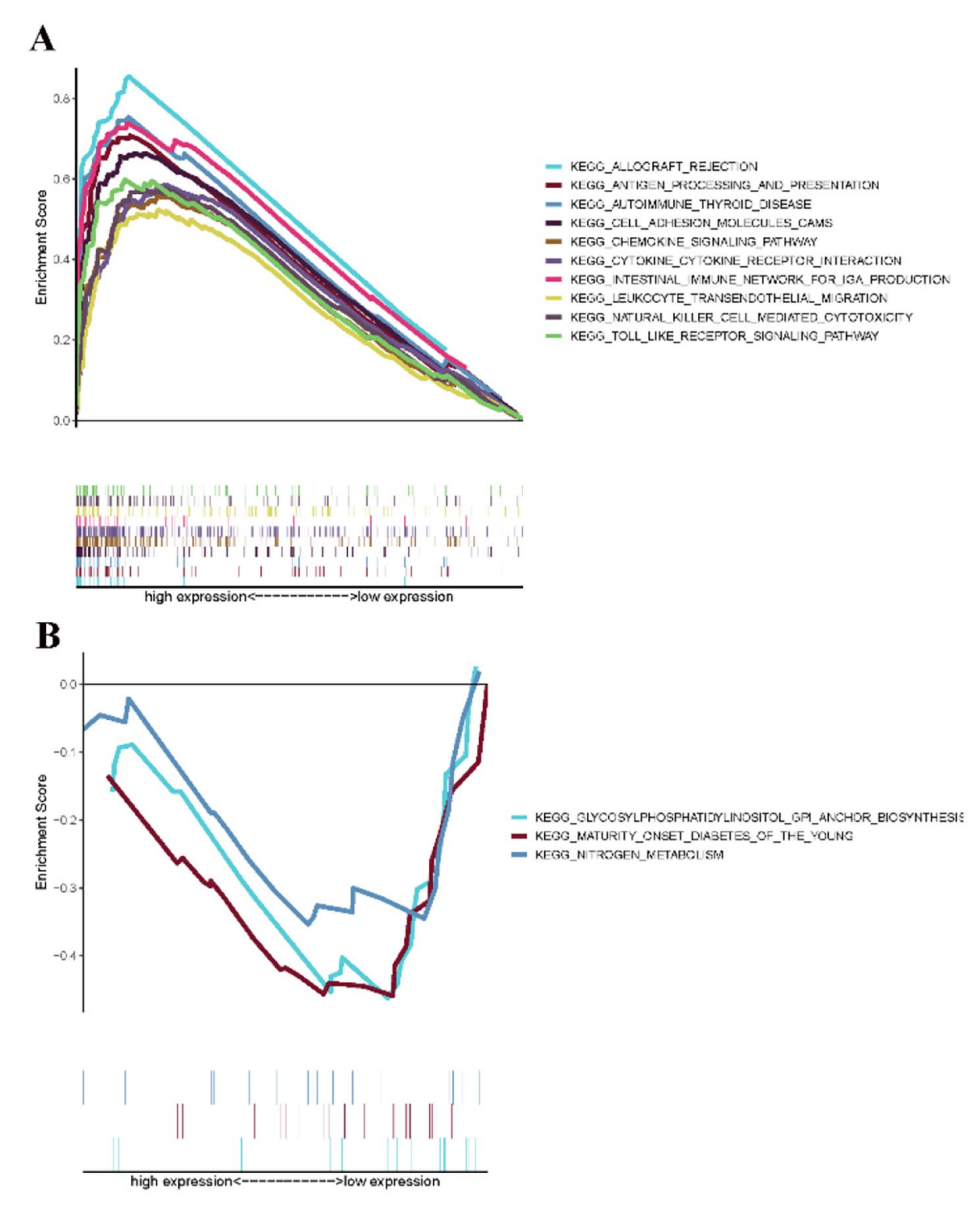
GSEA for samples with high CXCL10 expression and low expression. (A) GSEA for samples with high CXCL10 expression; (B) GSEA for samples with low CXCL10 expression

Compared with the median level of CXCL10 expression, GSEA was completed in the CXCL10 high-expression and low-expression groups. As shown in Fig. 7A, genes in the CXCL10 overexpression group are rich in immune-related activities, such as allograft rejection, cell adhesion, chemokine signaling, and Toll-like receptors. As shown in Fig. 7B, genes in the CXCL10 low-expression group were mainly enriched in metabolic pathways, such as nitrogen metabolism, maturity onset diabetes, and glycosylphosphatidylinositol. These results suggest that CXCL10 may be a potential indicator of TME status, in which metabolic-related activities are transformed into immune-related activities.

### Correlation of CXCL10 with the proportion of TICs

**Fig. 8:**
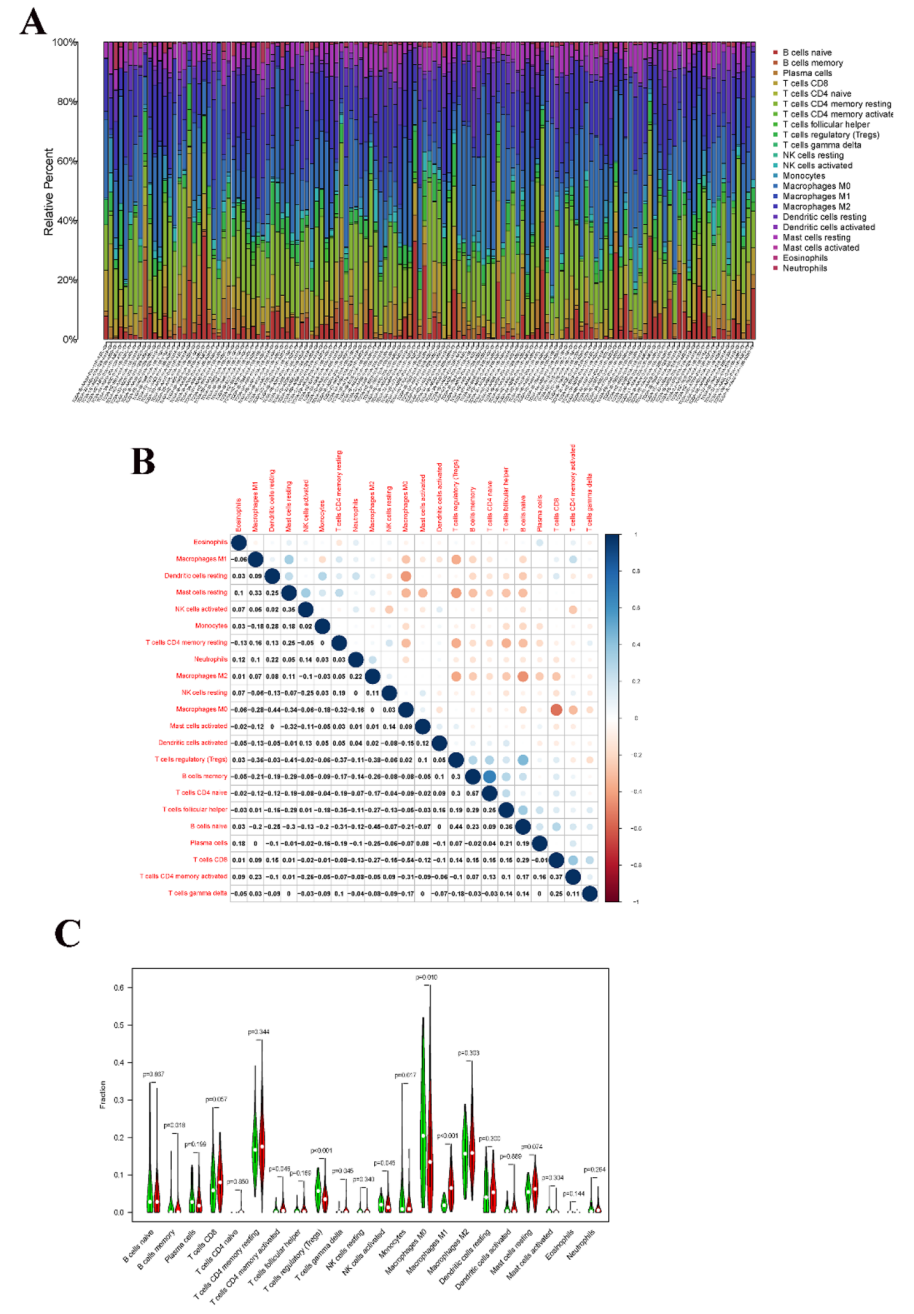
TIC profile in tumor samples and correlation analysis. (A) Barplot showing the proportion of 21 kinds of TICs in PAAD samples; (B) Heatmap showing the correlation between 21 kinds of TICs and numeric; (C) Violin plot showed the ratio differentiation of 21 kinds of immune cells between PAAD samples with high CXCL10 expression and low CXCL10 expression

To further confirm the correlation between CXCL10 expression and the immune microenvironment, the proportion of tumor-infiltrating immune subsets was analyzed using the CIBERSORT algorithm, and 21 kinds of immune cell profiles in PAAD patients were assessed (Fig. 8A). There were significant correlations between some kinds of cells (Fig. 8B), such as macrophages M0 and dendritic cells resting, T cells CD4 naïve and B cells memory, B cells naïve and macrophages M2. According to the median expression level of CXCL10, the samples were divided into high and low subgroups, and the results showed that there were significant differences among the 7 kinds of cells, including memory B cells (*P* = 0.018), CD4 memory activated T cells (*P* = 0.046), regulatory T cells (Tregs) (*P* < 0.001), gamma delta T cells (*P* = 0.045), activated NK cells (*P* = 0.045), M0 macrophages (*P* = 0.010), and M1 macrophages (*P* < 0.001) (Fig. 8C).Additionally, there were significant correlations between CXCL10 expression and the proportion of memory B cells (*r*=-0.24, *P* = 0.0046), CD4 memory activated T cells (*r* = 0.25, *P* = 0.0041), Tregs (*r* = 0.36, *P =* 0.00021), gamma delta T cells (*r*=-0.24, *P* = 0.0046), activated NK cells (*r*=-0.21, *P* = 0.0085), M0 macrophages (*r*=-0.29, *P* = 0.0008), and M1 macrophages (*r* = 0.277, *P* < 0.0001). These results further support the effect of CXCL10 expression on the immune activity of the TME.

## Discussion

In the current study, we attempted to identify TME-related genes that affect the survival and TNM classification of PAAD patients from the TCGA database. First, based on the DEGs between the lower and higher immune score and stromal score groups, a total of 772 TME-related genes were identified. Then, CXCL10 (IP-10) was identified to be involved by intersection analysis of the PPI network and univariate Cox regression. The gene expression of CXCL10 was correlated with TNM classification and survival by the TCGA and verification databases. Finally, the expression of CXCL10 might be an indicator of TME status by GSEA and has a significant correlation with the proportion of TICs.

The TME plays an important role in the occurrence and development of PAAD and is the main reason for the insensitivity to radiotherapy and chemotherapy and the poor prognosis of patients with PAAD [2, 11, 12]. As many mechanisms to suppress immune responses during the development of PAAD and the immunosuppressive microenvironment have evolved, the activation and function of immune cells in the TME have been limited, thus greatly affecting the effectiveness of treatment [13]. It is of great clinical significance to explore potential therapeutic targets based on TME remodeling and promote the transformation of the TME from tumor friendly to tumor suppressor. The TME of PAAD is mainly composed of three parts, namely, matrix cell components, immune cells, and soluble cytokines [5]. Even if there was no significant difference, the results showed that the immune score and stromal score of patients with relatively advanced tumors were higher than those of patients with relatively early tumors (Fig. 1).

It was found that the expression of CXCL10 was closely related to 7 kinds of TICs by the CIBERSORT algorithm, including memory B cells, CD4 memory activated T cells, Tregs, gamma delta T cells, activated NK cells, M0 macrophages, and M1 macrophages. There were many types of immune cells in the TME of PAAD, but most of these were imbalanced in quantity and function [6]. A previous study indicated that the number of CD8 + T cells was significantly reduced and the number of Treg cells was significantly increased with an increase in the degree of malignancy of PAAD [14]. The gene expression of CXCL10 was positively corrected with Tregs and negatively corrected with CD8 + T cells in this study. The results show that there were correlations between CXCL10 and Tregs and CD8 + T cells. CCL22 is secreted by PAAD cells, and chemotactic Tregs accumulate to the tumor site by binding the CCR4 receptor of Tregs [15]. Inhibitory cytokines, such as TGF-β and IL-10, are secreted by Tregs in the TME, or cell contact-dependent protein pathways, such as programmed death-1 (PD-1), are activated and then inhibit the activation and function of T cells, which mediates the immune tolerance of T cells to PAAD-associated antigens so that tumor cells escape immune surveillance [7, 15].

Affected by the suppressive TME of PAAD, the number of NK cells was significantly reduced, and studies have shown that the expression level of NK cell surface activation receptors (such as NKG2D) is negatively correlated with the degree of PAAD [16]. However, it was found that the number of activated NK cells in patients with high CXCL10 expression was high. The results were inconsistent with the expected results and need further study. The immunosuppressive factors in the TME of PAAD, such as TGF-β and IL-10, can stimulate macrophages to differentiate into M2 macrophages with protumor effects and lead to a decrease in M1 macrophages with tumor-suppressor effects [17]. In this study, patients with high expression levels of CXCL10 showed relatively advanced tumors and showed a decline in M1 macrophages; however, there was no significant difference compared to M2 macrophages. A study suggested that the migration of mast cells (MCs) into the TME leads to local immunosuppression, which facilitates the development and metastasis of PAAD, and suggested that targeting the immunosuppressive function of MCs may be a new treatment strategy for PAAD [18]. Of course, the expression of bone marrow mesenchymal stem cells also plays the important role in the progression of PAAD, and IL-6 produced by bone marrow mesenchymal stem cells plays a key role [19]. A study has indicated that bone marrow mesenchymal stem cells derived exosomes carrying MiR-124 can inhibit the proliferation of pancreatic cancer cells by co-culture of cancer cells with bone marrow mesenchymal stem [20]. In the presence of ganciclovir, HSV-Tk transfected MSCs significantly reduced the growth and metastasis rate of PAAD [21]. However, due to the limited sample size, there was no significant difference in comparing MCs.

The final screened gene, CXCL10, was correlated with TNM classification and survival in the TCGA and verification databases. In accordance with the results of previous studies, the expression of IP-10 was upregulated in tumor tissue compared with normal pancreatic tissue, and its expression was also associated with poor survival [22]. CXCL10, also called interferon-induced protein 10 (IP-10), is stimulated by IFN-γ in a dose-dependent and time-dependent manner [23]. Chemokine CXC receptor 3 (CXCR3) is the only receptor of CXCL10. The expression of CXCR3 is relatively lower on T cells, B cells, and monocytes in the resting phase; however, CXCR3 is mainly expressed on the surface of T cells, B cells and NK cells in the activation phase and has the function of promoting tumor proliferation [24]. Although there is some controversy in terms of the promotion or inhibition of tumors, more studies have indicated that the CXCL10/CXCR3 axis plays an important role in the development of various tumors [8]. Pancreatic stellate cells in PAAD can secrete CXCL10, which can recruit CXCR3^+^CD4^+^ cells, CXCR3^+^CD8^+^ cells and CXCR3^+^ Tregs into tumor tissue. The function of cytotoxic T lymphocytes (CTLs) or NK cells is blocked by Tregs and produces an immunosuppressive environment, which leads to tumor progression [25]. Therefore, the promotion or inhibition effect of CXCL10 on tumor immunity may depend on the balance between CXCR3^+^CD8^+^ cells and CXCR3^+^ Tregs. The peripheral blood mononuclear cells (PBMCs) of PAAD patients contained more Tregs than those of healthy volunteers, indicating that CXCL10 may preferentially recruit circulating Tregs into PAAD compared to other subtypes of T cells [26]. CXCL10/CXCR3 mainly activates downstream signaling pathways (including MAPK and PI3K/Akt) and then produces biological effects, such as promoting angiogenesis and inhibiting tumor-related immunity.

The enrichment activities in the CXCL10 high-expression group and CXCL10 low-expression group were different by GSEA and indicated that the expression of CXCL10 might be an indicator of the conversion of TME status from immune-dominant to metabolic-dominant. As expected, the immune-related pathways were enriched in the high-expression group. Interestingly, the metabolic pathway was enriched in the CXCL10 low-expression group. It has been reported that the K-ras mutation in PAAD could lead to the transformation of glucose metabolism, resulting in the production of large amounts of lactate and the decrease in the pH value of the TME [9].

The study has several limitations. Firstly, the main focus of this study is bioinformatics analysis based on the TCGA database, and functional experiments are needed to uncover the predictive mechanisms of CXCL10. Secondly, the confounding effects of treatment factors differed from those of the control group due to lack of treatment information. Finally, more independent cohorts are needed to extend our model to other populations, especially patients with advanced PAAD[27, 28].

In conclusion, CXCL10 is a prognostic indicator of the development and survival of PAAD patients and may mark the transformation of TME status from immunological to metabolic advantage. CXCL10 expression was significantly correlated with the proportion of TICs, especially activated CD4 memory T cells, Tregs, gamma delta T cells, activated NK cells, M0 macrophages and M1 macrophages. Our characteristics may reflect TME disorders and provide potential biomarkers for metabolic therapy and prediction of treatment response. However, validation of this risk in more independent functional experiments is need.

## Electronic supplementary material

Below is the link to the electronic supplementary material.

Supplementary Material 1

Supplementary Material 2

## Data Availability

The datasets of TCGA can be found in online repositories (https://portal.gdc.cancer.gov/), and the other data used to support the findings of this study are available from the corresponding author upon request.
