# Supplementary material for: CXCL10 is a prognostic marker for pancreatic adenocarcinoma and tumor microenvironment remodeling
Source: BMC Cancer. 2023 Feb 13;23:150. doi: 10.1186/s12885-023-10615-w (PMC9926744; doi:10.1186/s12885-023-10615-w)
Supplement: Supplementary file 1 — Supplementary Material 1 [file 12885_2023_10615_MOESM1_ESM.docx]

Supplementary Table 1. Characteristics of PAAD Patients from TCGA database and Local hospital

| Variables | PAAD patients of TCGA database | PAAD patients of local hospital |
| --- | --- | --- |
| n | 182 | 22 |
| Male, n (%) | 99 (54.4%) | 16 (72.7%) |
| Female, n (%) | 83 (45.6%) | 6 (27.3%) |
| Age, median (IQR) | 65 (57-73) | 68 (56.5-75) |
| Grade, n (%) |  |  |
| G1 | 32 (18.6%) | 5 (22.7%) |
| G2 | 97 (53.3%) | 14 (63.6%) |
| G3 | 51 (28.0%) | 2 (9.1%) |
| G4 | 2 (1.1%) | 1 (4.5%) |
| Stage, n (%) |  |  |
| Stage I | 20 (11.0%) | 3 (13.6%) |
| Stage II | 150 (82.4%) | 6 (27.3%) |
| Stage III | 4 (2.2%) | 9 (40.9%) |
| Stage IV | 5 (2.7%) | 4 (18.2%) |
| T stage, n (%) |  |  |
| T1 | 6 (3.3%) | 2 (9.1%) |
| T2 | 24 (13.2%) | 7 (31.8%) |
| T3 | 146 (80.2%) | 11 (50%) |
| T4 | 4 (2.2%) | 2 (9.1%) |
| Unknown | 2 (1.1%) |  |
| Metastasis, n (%) |  |  |
| No | 83 (45.6%) | 17 (77.3%) |
| Yes | 4 (2.2%) | 5 (22.7%) |
| Unknown | 95 (52.2%) |  |
| Lymph node, n (%) |  |  |
| N0 | 49 (26.9%) | 14 (63.6%) |
| N1 | 128 (70.3%) | 8 (36.4%) |
| Unknown | 5 (2.7%) |  |

PAAD: pancreatic adenocarcinoma; TCGA: The Cancer Genome Atlas; IQR: interquartile range.
