# Supplementary material for: CXCL10 is a prognostic marker for pancreatic adenocarcinoma and tumor microenvironment remodeling
Source: BMC Cancer. 2023 Feb 13;23:150. doi: 10.1186/s12885-023-10615-w (PMC9926744; doi:10.1186/s12885-023-10615-w)
Supplement: Supplementary file 2 — Supplementary Material 2 [file 12885_2023_10615_MOESM2_ESM.docx]

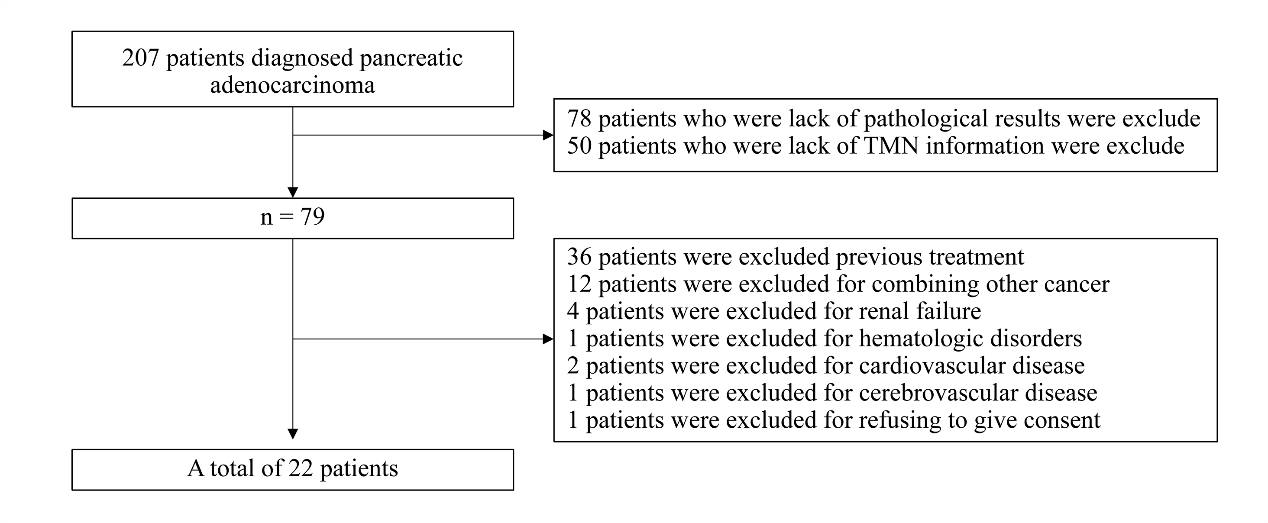


**Supplementary Figure 1.** Study cohort flow chart of local hospital patients.


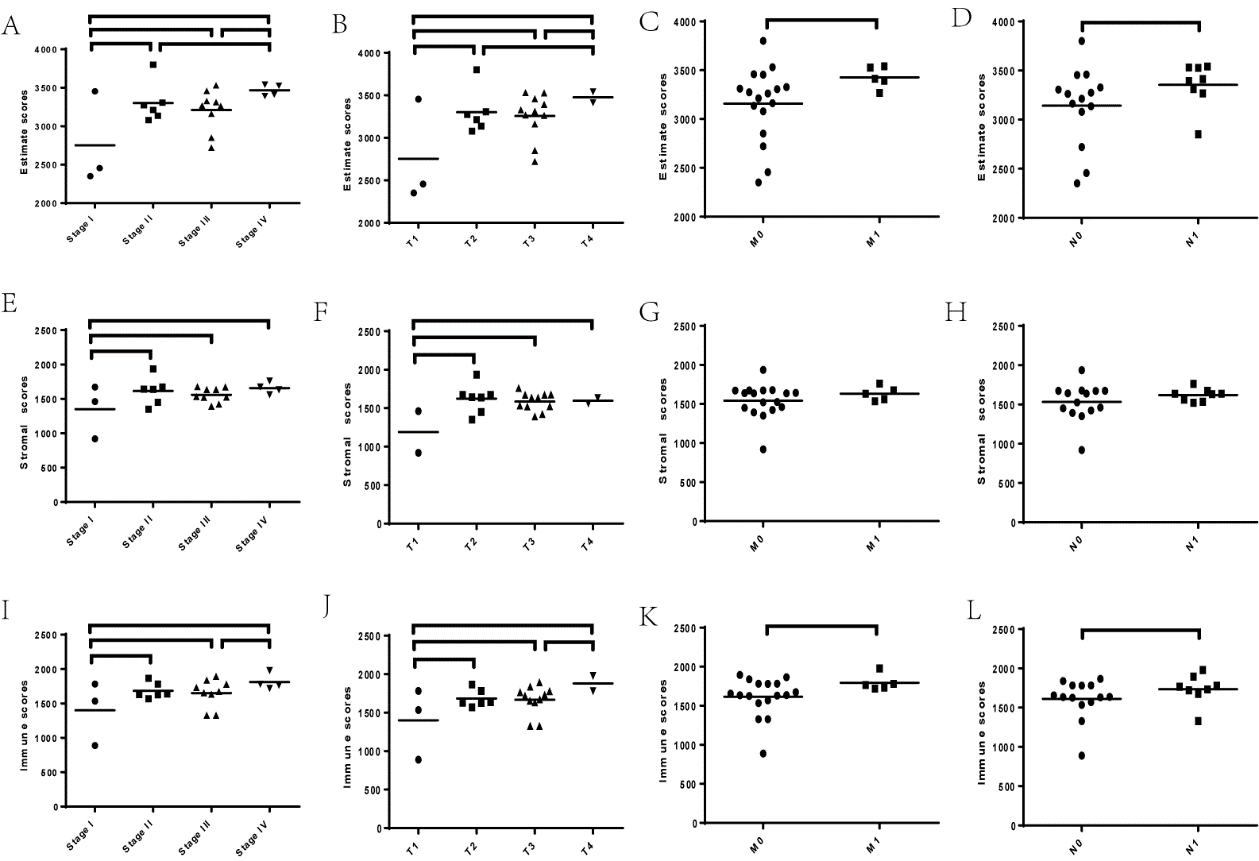


**Supplementary Figure 2.** Correlation of ESTIMATE Score, Immune Score and Stromal Score with clinical characteristics in local hospital databases. (A–D) Distribution of ESTIMATE Score in different stage and TMN classification; (E–H) Distribution of Immune Score in different stage and TMN classification; (I–L) Distribution of Stromal Score in different stage and TMN classification.
